# Effect of deposition temperature on the structural and optical properties of chemically prepared nanocrystalline lead selenide thin films

**DOI:** 10.3762/bjnano.3.50

**Published:** 2012-06-06

**Authors:** Anayara Begum, Amir Hussain, Atowar Rahman

**Affiliations:** 1 Department of Physics, Gauhati University, Guwahati-781014, Assam, India

**Keywords:** chemical bath deposition, lattice parameter, lead selenide, Nelson–Riley plot, optical absorption

## Abstract

Nanocrystalline lead selenide (PbSe) thin films were prepared on glass substrates by a chemical bath deposition method, using sodium selenosulfate (Na_2_SeSO_3_) as a source of Se^2−^ ions, and lead acetate as a source of Pb^2+^ ions. Trisodium citrate (TSC) was used as a complexing agent. PbSe films were prepared at various deposition temperatures while the pH value was kept fixed at 11, and the effect on the resulting film properties was studied by X-ray diffraction (XRD), X-ray fluorescence (XRF), scanning electron microscopy (SEM) and optical absorption studies. The structural parameters, such as the lattice constant (*a*), crystallite size (*D*), dislocation density (ρ) and microstrain (ε) were evaluated from the XRD spectra. It was found that average crystallite size, as calculated from Scherrer’s formula, increased from 23 to 33 nm as the deposition temperature was varied from 303 to 343 K. The dislocation density and microstrain were found to vary inversely with the crystallite size, whereas the lattice constant was found to increase with an increase in crystallite size. The optical absorption spectra of the nanocrystalline PbSe films showed a blue shift, and the optical band gap (*E*_g_*)* was found to increase from 1.96 to 2.10 eV with the decrease in crystallite size.

## Introduction

Lead selenide (PbSe) with its narrow band gap (*E*_g_ = 0.27 eV) [1] is an important semiconductor of the IV–VI lead chalcogenides group. PbSe has a large exciton Bohr radius (46 nm), which offers the opportunity to achieve strong size quantization at relatively large crystal size [2–3]. PbSe has wide applications in long and mid-wavelength infrared detectors, optical amplifiers, mid-infrared lasers, as thermoelectric materials, and as Pb^2+^ ion selective sensors [4–7]. Among the various techniques used to prepare PbSe thin films, such as vacuum evaporation [8], microwave heating [9], pulsed laser deposition [10], electrochemical atomic layer epitaxy [11], and electrodeposition [12], the chemical bath deposition method [13–14] is relatively simple and cost-effective, and has the advantage that it allows control over deposition parameters such as the pH, the concentration of ions, deposition temperature, etc., with great ease.

In this paper we report the preparation of nanocrystalline PbSe thin films by the chemical bath method at different deposition temperatures and at a fixed pH value, and we discuss the dependence of various structural and optical properties on the deposition temperature.

## Experimental

Lead selenide thin films were deposited by using lead acetate (Pb(CH_3_COO)_2_) and freshly prepared sodium selenosulfate (Na_2_SeSO_3_) as Pb^2+^ and Se^2−^ ions source, respectively, in the presence of trisodium citrate (TSC) as a complexing agent. The Na_2_SeSO_3_ solution was prepared by mixing 3 g selenium powder with 30 g anhydrous sodium sulfite in 150 ml of distilled water under constant stirring for 8–10 h at 353 K [15].

To prepare thin films of PbSe, 0.5 M Pb(CH_3_COO)_2_ solution was prepared. To this solution was added 1 M TSC solution, and then the pH of the solution was adjusted to 10.80 by drop-wise addition of KOH. Finally, Na_2_SeSO_3_ was added and the pH of the final deposition bath was adjusted to 11. The glass substrates were vertically immersed in the deposition bath at the desired temperature. After a deposition period of 2 h, the substrates were taken out, rinsed in distilled water and dried. Four different sets of PbSe films were prepared at different temperatures (328, 333, 338, and 343 K). The as-deposited films were uniform, continuous, and pinhole free. These were specularly reflective, and extremely adherent to the substrates. However the PbSe films prepared at room temperature (303 K) were found to be pale brown in appearance.

The X-ray diffraction patterns of the as-deposited PbSe thin films were taken on an X-ray diffractometer (XPERT PRO Philips) at room temperature. Studies of the surface morphology of the chemically deposited PbSe films were achieved by scanning electron microscope (JEOL JSM 6360).

The X-ray fluorescence study (XRFS) was performed on an AXIOS spectrometer (DY 840) for elemental analysis of the as-prepared films and optical absorption studies were carried out using a UV–vis spectrophotometer (VARIAN CARY 300 scan) in the wavelength range 360–900 nm. The thickness of the PbSe thin films was measured by the multiple beam interferometer technique.

## Results and Discussion

### Film growth

A PbSe thin film is formed when the ionic product of Pb^2+^ and Se^2−^ ions exceeds the solubility product of PbSe (≈10^−38^ at 300 K) [16]. The deposition process is based on the slow release of Pb^2+^ and Se^2−^ ions in the solution. The complexing agent trisodium citrate controls the Pb^2+^ concentration and slowly releases Pb^2+^ ions into the solution. The proposed reaction mechanism for formation of PbSe thin films is as follows,

[1]
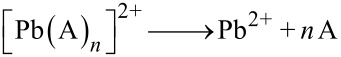


[2]
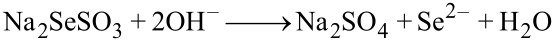


[3]
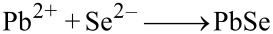


where A is trisodium citrate.

### X-ray diffractograms

**Figure 1:**
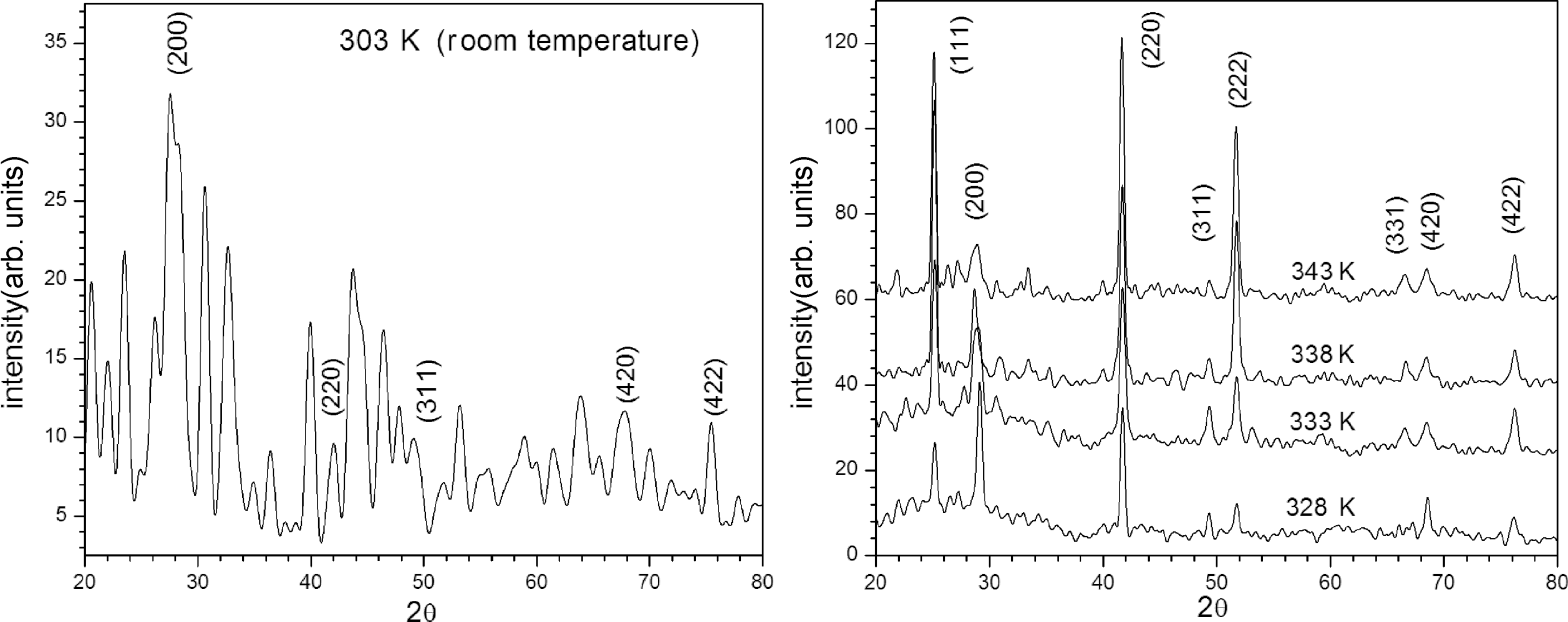
X-ray diffraction patterns of PbSe thin films prepared at different temperatures.

Typical XRD patterns obtained from films grown at different temperatures and at pH 11 are shown in Figure 1. The observed “*d*” spacings and the respective prominent peaks correspond to reflections of the (111), (200), (220), (311), (222), (331), (420) and (422) planes and are in good agreement with the standard data (JCPDS No. 06-0354). Thus, the XRD pattern reveals the polycrystalline nature of the as-deposited PbSe thin films with cubic structure.

The most intense peak for each of the samples prepared at 328, 333, 338, and 343 K corresponds to (200), (111), (111) and (220), respectively. This indicates that the orientation of the grain growth for PbSe films prepared at different temperatures is along different directions. The XRD pattern of PbSe prepared at room temperature (303 K) shows peaks for (200), (220), (420) and (422) in addition to several other peaks, which are mainly due to impurity phases (such as Pb, Se, PbO, or other Pb compounds).

### Crystallite size

**Figure 2:**
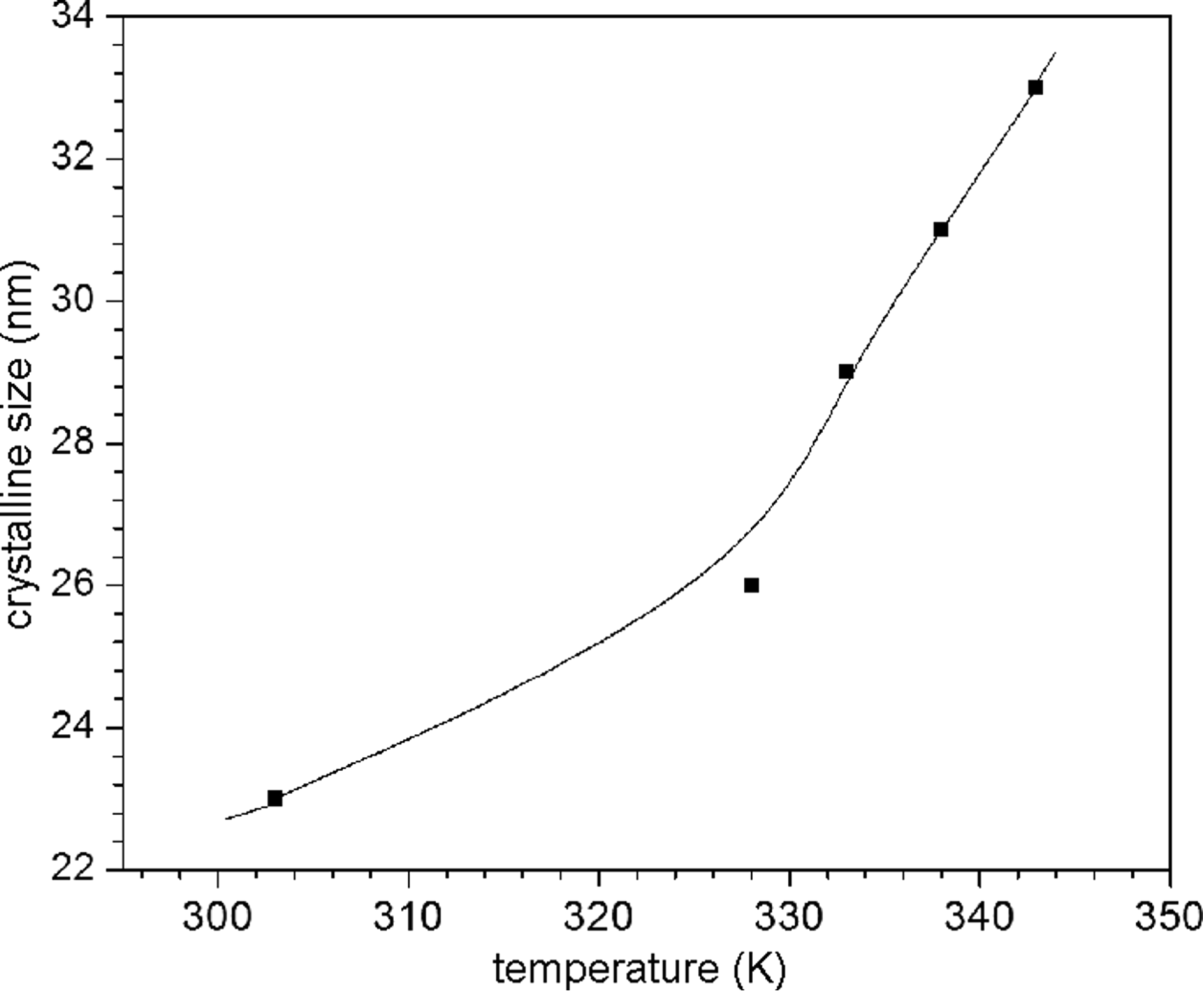
Variation of the average crystallite size of PbSe thin films with the deposition temperature.

The average crystallite size of PbSe thin films prepared at different temperatures calculated using Scherrer’s formula [17] was found to increase from 23 to 33 nm with an increase of temperature from 303 to 343 K, as shown in Figure 2. The rate of the deposition reaction increases at higher temperature and the crystallites grow faster resulting in a larger size.

### Lattice constant

The lattice parameter “*a*” for cubic structure was determined by using the relation

[4]
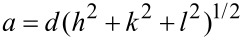


where “*d*” is the spacing between the planes in the atomic lattice, and (*hkl*) are the Miller indices. The lattice constant values are found to be slightly different for different orientations of the same film. The probable explanation for this was given by Mothura et al. [18]. Therefore, corrected values of the lattice constants were determined from the intercept of the Nelson–Riley plots. The Nelson–Riley curve is plotted between the calculated “*a*” for different planes and the error function [19]

**Figure 3:**
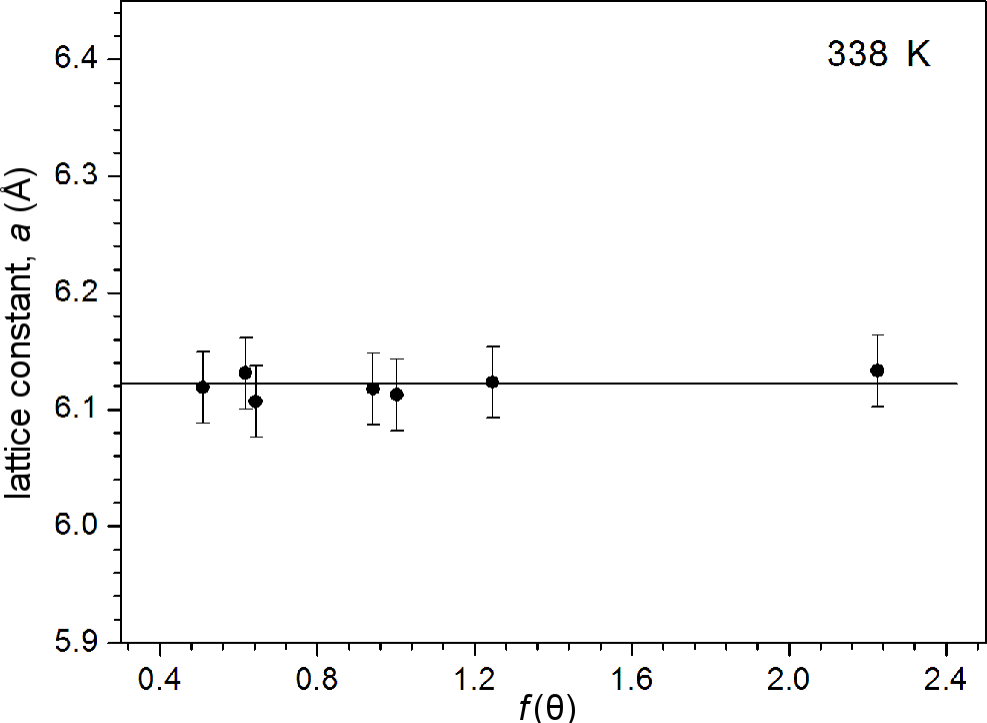
Nelson–Riley plot for PbSe thin film prepared at 338 K.

[5]
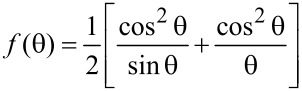


and the corrected value of “*a*” is obtained by extrapolating the plot to θ = 90°. A typical Nelson–Riley plot for a PbSe thin film is shown in Figure 3.

The lattice parameter is observed to increase slightly from 6.112 to 6.129 Å with deposition temperature. All the values of lattice constant of the as-prepared PbSe thin films were found to be different from the values of the bulk material (6.124 Å, JCPDS 06-0354). The deviation in the values of the lattice constant of the as-prepared PbSe films from the bulk value indicates the presence of strain in the films. The strain in the prepared PbSe films may arise due to the change of lattice nature and concentration of native imperfections during the film formation.

### Dislocation density (δ) and microstrain (ε)

The dislocation density (δ) was calculated from Williamson and Smallman’s formula [20]

[6]
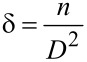


where “*n*” is a factor, which when equal to unity gives the minimum dislocation density, and “*D*” is the average crystallite size. The average microstrain (ε) developed in the as-prepared PbSe films was calculated by using the relation [21]

[7]
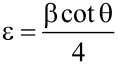


where “β” is the full width at half maximum and θ is the Bragg angle.

**Figure 4:**
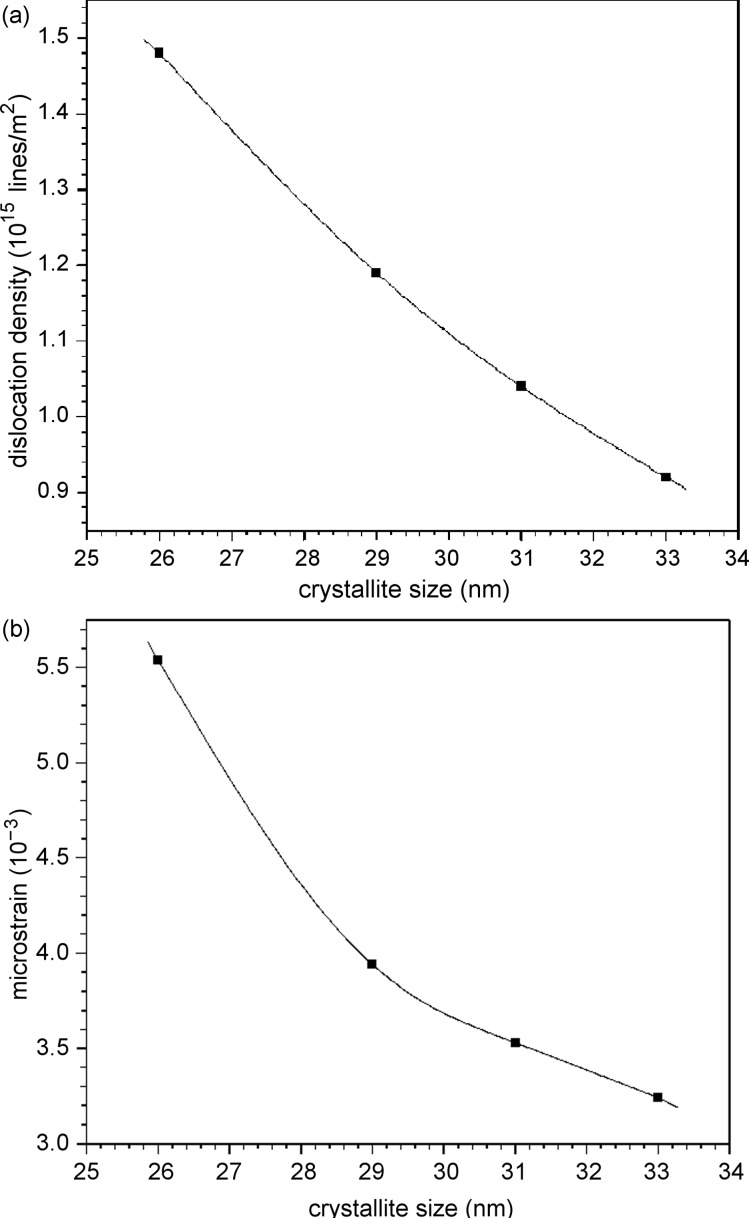
Variation of (a) dislocation density and (b) microstrain with crystallite size of the PbSe thin films.

It has been observed that the dislocation density and microstrain decrease with increase in crystallite size, as shown in Figure 4, which indicates a lower number of lattice imperfections. This may be due to a decrease in the occurrence of grain boundaries because of an increase in the crystallite size of the film with increasing temperature.

### Surface morphology

**Figure 5:**
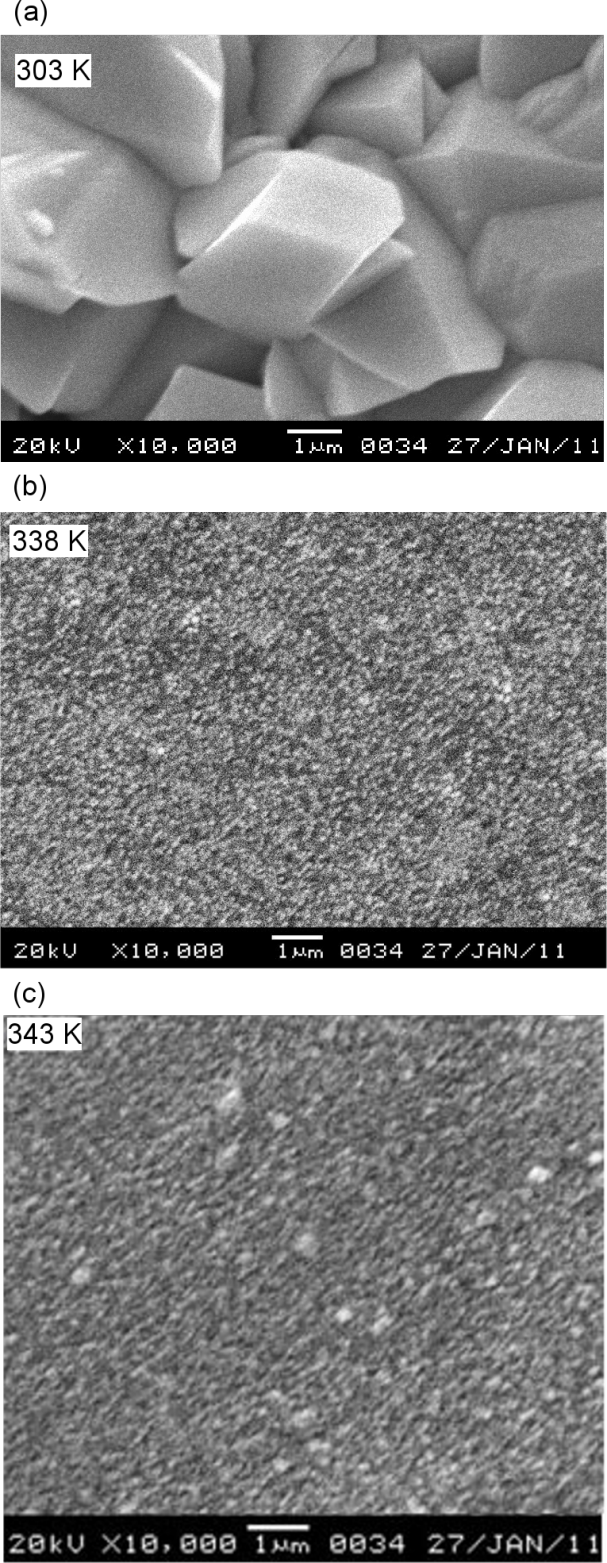
SEM micrograph of PbSe prepared at (a) 303 K (b) 338 K and (c) 343 K.

Scanning electron microscopy gives valuable information regarding the shape and size of the grains on the surface of the deposited thin films. Figure 5a shows the SEM image of PbSe thin films prepared at 303 K (room temperature), and reveals cube-like structures, which are aggregated. From the SEM image of PbSe prepared at 338 K (Figure 5b) and 343 K (Figure 5c), it was observed that the PbSe thin films consisted of spherical grains and were homogenous, without any voids or cracks.

The crystallite size obtained by XRD is equivalent to the mean size of the domains that scatter X-rays coherently [22]. The grain size measured from SEM images is the surface morphology of grains that are agglomerated crystallites, leading to larger values of grain size.

### X-ray fluorescence (XRF) studies

**Figure 6:**
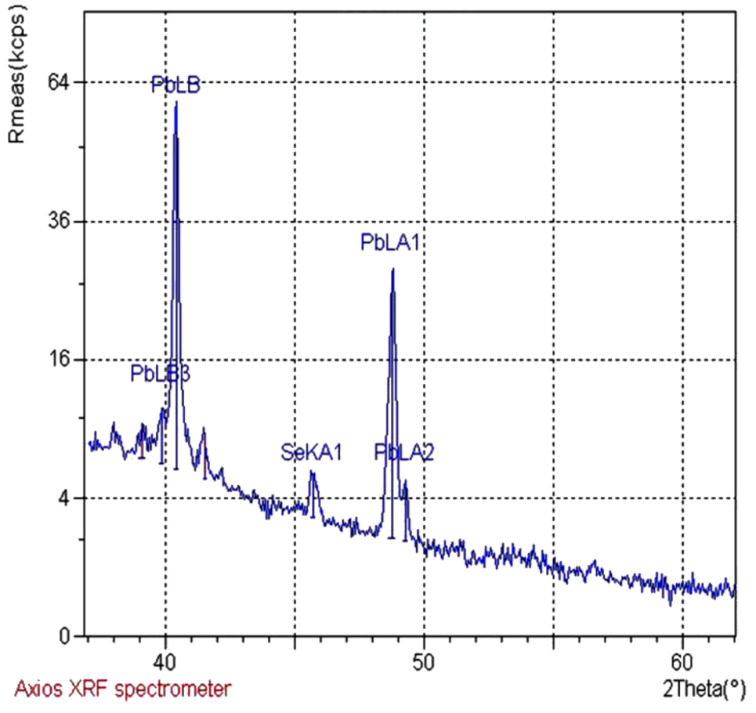
XRF spectra of PbSe prepared at 333 K.

Figure 6 shows the XRF spectra of PbSe thin films prepared at 333 K. The spectrum exhibits prominent peaks of the Pb Lβ, Pb Lα_1_, Pb Lα_2_ and Se Kα_1_ lines showing the presence of Pb and Se in the prepared films.

### Optical properties

**Figure 7:**
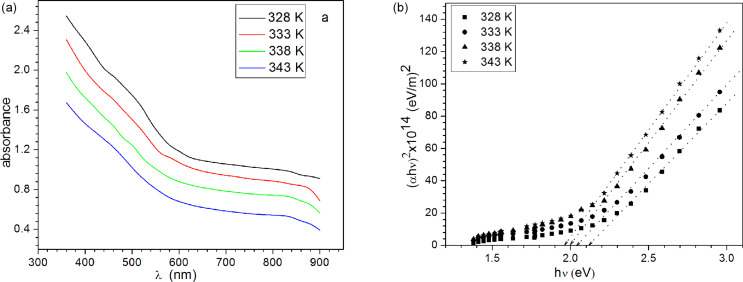
(a) UV absorption spectra; (b) (α*h*ν)^2^ vs (*h*ν) plots of PbSe thin films.

The absorption spectra of PbSe thin films recorded at room temperature as a function of wavelength in the range 360–900 nm, is shown in Figure 7a. It shows that the optical absorption of PbSe thin films increases with the deposition temperature. This may be attributed to the increase in crystallite size and decrease in defects. The (α*h*ν)^2^ versus (*h*ν) plots of PbSe thin films are linear over a wide range of photon energies, as shown in Figure 7b. This indicates the presence of a direct optical band gap in the as-prepared PbSe thin films [23]. The optical band gap of these films was obtained by extrapolating the linear portion of the curve to the energy axis.

**Figure 8:**
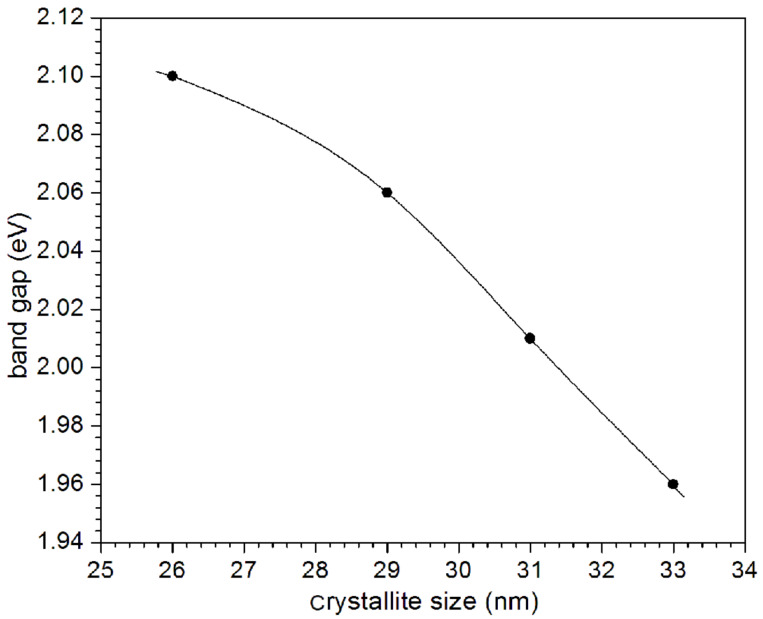
Variation of the band gap with crystallite size of PbSe thin films.

The band gap so obtained was observed to decrease from 2.10 to 1.96 eV, as the deposition temperature was increased from 328 to 343 K. Typical variation of band gap with crystallite size in the nanocrystalline PbSe thin films is shown in Figure 8, which indicates an increase in the band gap with a decrease in crystallite size (the structural parameters and band-gap energies are also summarized in Table 1). Clearly, the observed values of *E*_g_ are higher than the value of the bulk optical band gap of PbSe [0.27 eV] [1] due to quantum confinement in the nanocrystalline PbSe thin films. Similar changes in the band gap energy “*E*_g_” for PbSe thin films with smaller crystallite sizes have been reported for chemically deposited PbSe thin films by Gorer et al. [24]. The value of the band gap was found to vary from 0.55 to 1.55 eV, depending on the crystallites size, by Gorer et al.

**Table 1:** Structural parameters and band-gap energies of PbSe thin films deposited at different temperatures.

Sl no.	deposition temperature (K)	average crystallite size (nm)	lattice constant (Å)	microstrain (ε) × 10^−3^	dislocation density (δ) (× 10^15^ lines/m^2^)	band gap (*E*_g_) from absorption spectra (eV)

1	328	26	6.112	5.54	1.48	2.10
2	333	29	6.121	3.94	1.19	2.04
3	338	31	6.122	3.53	1.04	2.00
4	343	33	6.129	3.24	0.92	1.96

## Conclusion

Nanocrystalline PbSe thin films have been successfully deposited at different temperatures (328 to 343 K) above room temperature (303 K). The grains were found to be spherical in shape and the crystallite sizes found from XRD increased from 26 to 33 nm as the deposition temperature was increased from 328 to 343 K. The optical band gap of the PbSe films was found to increase from 1.96 to 2.10 eV with the decrease in crystallite size, which is in accordance with the quantum confinement effect. The dislocation density and microstrain were found to decrease with increase in deposition temperature and, hence, crystallite size, indicating the improvement of PbSe thin films.
